# Supplementary material for: Early-twentieth-century cold bias in ocean surface temperature observations
Source: Nature. 2024 Nov 20;635(8039):618–24. doi: 10.1038/s41586-024-08230-1 (PMC11578896; doi:10.1038/s41586-024-08230-1)
Supplement: Supplementary file 1 — This file contains Supplementary Text, Figs, 1–8 and Tables 1–4. The Supplementary Text covers supplementary analysis and discussion on the evaluation of reconstruction methods, individual palaeoclimate proxies and SST data contributing to the ICOADS database. Supplementary Tables provide an overview of all reconstructions, source datasets and model simulations. [file 41586_2024_8230_MOESM1_ESM.pdf]

nature portfolio

https://doi.org/10.1038/s41586-024-08230-1

Supplementary information

Early-twentieth-century cold bias in ocean surface temperature observations

In the format provided by the authors and unedited

Supplementary Information to ‘Early-twentieth-century cold bias in ocean surface temperature observations’

Sebastian Sippel[1,2], Elizabeth C. Kent[3], Nicolai Meinshausen[4], Duo Chan[5], Christopher Kadow[6], Raphael Neukom[7,8], Erich M. Fischer[2], Vincent Humphrey[9], Robert Rohde[10], Iris de Vries[2], Reto Knutti[2]

[1]*Institute for Meteorology, Leipzig University, 04103 Leipzig, Germany*

[2]*Institute for Atmospheric and Climate Science, ETH Zürich, 8006 Zürich, Switzerland*

[3]*National Oceanography Centre, Southampton SO14 3ZH, United Kingdom*

[4]*Seminar for Statistics, ETH Zürich, 8006 Zürich, Switzerland*

[5]*School of Ocean and Earth Science, University of Southampton, Southampton, United Kingdom*

[6]*Deutsches Klimarechenzentrum GmbH, 20146 Hamburg, Germany*

[7]*WSL Institute for Snow and Avalanche Research SLF, Davos, Switzerland*

[8]*Climate Change, Extremes and Natural Hazards in Alpine Regions Research Centre, CERC, Davos, Switzerland*

[9]*Federal Office of Meteorology and Climatology MeteoSwiss, 8059 Zürich-Airport, Switzerland*

[10]*Berkeley Earth, Berkeley, CA, USA*

Contents

1 Evaluation of reconstruction method 5

2 Supplementary Reconstructions 10

3 Analysis of individual paleoclimate proxies from terrestrial and marine sources 19

4 Supplementary analysis of ICOADS contributing sources 20

5 Ocean warming constrained by land warming and paleoclimate reconstructions using 50-year trends 27

6 Supplementary Tables 28

List of Supplementary Figures

1 Evaluation of annual GMST reconstruction as a function of time and training setup 9

2 Global mean surface temperature (GMST) reconstruction from the land and ocean record, but without including biases and uncertainties in the training setup. 13

3 Global mean sea surface temperature (GMSST) reconstruction from the land and ocean record. 15

4 Global mean land surface air temperature (GMLSAT) reconstruction from the land and ocean record. 17

5 GMST reconstruction from SSTs with global mean removed 18

6 Dupelim output information from ICOADS Release 1, 1800-1899 25

7 Dupelim output information from ICOADS Release 1, 1900-1945 26

8 Ocean warming constrained by land warming and paleoclimate reconstructions using 50-year trends 27

List of Supplementary Tables

1 Overview of global mean temperature reconstructions by source and training dataset. 28

2 Overview of gridded observational datasets used for reconstructions 29

3 Overview of GMST datasets and paleoclimate reconstructions 30

4 Overview of CMIP6 models used in the analysis. 31

1 Evaluation of reconstruction method

In this supplementary section, we provide a comprehensive evaluation and further discussion of our reconstruction method, which was outlined in the main text. Our approach addresses the measurement uncertainties and biases in historical temperature records by utilizing the uncertainty and bias estimates developed for the CRUTEM5 and HadSST4 datasets (Kennedy et al. 2019; Osborn et al. 2021; Morice et al. 2021). Specifically, we incorporate these models to refine our regression coefficients, denoted as $\hat{\beta}^{*\,\text{GMST}}_{\text{Land:1895-06}}$ for coefficients that account for uncertainties (that is, uncertainty estimates are added to CMIP6 model for training of the statistical model), compared to $\hat{\beta}^{\text{GMST}}_{\text{Land:1895-06}}$ for coefficients derived without consideration of these uncertainties and biases (that is, no uncertainties added during training). The subscript indicates the source data (land or ocean) and the respective time step of the mask (e.g., June 1895). The overall setup is illustrated in Extended Data Fig. 1.

Illustrative Maps of Regression Coefficients In Extended Data Fig. 2, we show maps of regression coefficients for an illustrative time step with sparse coverage in the early record (June 1895). These maps show coefficients for the land and ocean early coverage both without considering uncertainties ($\hat{\beta}^{\text{GMST}}_{\text{Land:1895-06}}$, panel a; $\hat{\beta}^{\text{GMST}}_{\text{Ocean:1895-06}}$, panel c) and coefficients when uncertainty and bias estimates are considered at training time ($\hat{\beta}^{*\,\text{GMST}}_{\text{Land:1895-06}}$, panel b; $\hat{\beta}^{*\,\text{GMST}}_{\text{Ocean:1895-06}}$, panel d). When training is based on CMIP6 models only ($\hat{\beta}^{\text{GMST}}_{\text{Land:1895-06}}$, that is, without considering uncertainty and bias estimates), the algorithm assigns large positive weights to tropical land grid cells, particularly islands and coastal stations, which thus exert a predominant influence on GMST estimates (Extended Data

Figure 2a). A similar behaviour is observed for coefficients over the ocean ($\hat{\beta}^{\text{GMST}}_{\text{Ocean:1895-06}}$, Extended Data Fig. 2c), where in particular coastal ocean grid cells and ocean grid cells that lie adjacent to large unobserved regions (e.g., tropical Pacific) show large positive weights. This behaviour may be understood from the fact that tropical regions are highly informative for global temperature estimates, and the pattern of regression coefficients is qualitatively consistent with other global temperature estimators, such as those similar to kriging (Cowtan et al. 2018).

However, this approach may not be ideal because observational uncertainties are not considered, and uncertainties are not equally distributed across space. In particular tropical grid cells in the early record are likely affected by large relative uncertainties and a relatively poor station density. When uncertainties are incorporated at training time (see details in methods in the main text), regression coefficients are distributed more evenly across the spatial domain for both land and ocean (Extended Data Fig. 2b,d). This behaviour thus reflects the trade-off to make use of the most informative grid cells in the reconstruction, but at the same time minimizing the effect of uncertainties. Overall, this methodological adjustment reduces the influence of individual, uncertain grid cells. The performance of our regression method for reconstructing GMST in CMIP6 models for the illustrative June 1895 ocean and land coverage is good and shown in Extended Data Fig. 3.

Systematic Evaluation of Reconstruction Error We systematically assess the error estimates across time by comparing our reconstruction method to the global temperature average estimator (Cowtan et al. 2018), a method similar to kriging. The evaluation is based on a reconstruction of CMIP6 models' GMST from a sparse coverage mask of either land or ocean. We evaluate the

reconstruction method where uncertainties are considered and not considered at training time (i.e., $\hat{\beta}^{\text{GMST}}_{\text{Land}}$, $\hat{\beta}^{*\,\text{GMST}}_{\text{Land}}$, pink vs. red lines in Supplementary Figure 1). Both methods are evaluated against CMIP6 models not used in training, and either based on the raw output of those CMIP6 models without adding uncertainty estimates (dashed lines in Supplementary Figure 1), and for a scenario where uncertainty estimates are added for evaluation (solid lines in Supplementary Figure 1). The benchmark GTA estimator (Cowtan et al. 2018) is adapted such that the global temperature average is obtained for land and ocean separately; and in a second step we scale the obtained averages in CMIP6 data to obtain a GMST estimate from land and ocean data separately. Including our benchmark GTA estimator (Cowtan et al. 2018), this yields in total six scenarios.

The land-based reconstructions indicate a sharp reduction in annual mean squared errors for all scenarios over time as coverage increases (Supplementary Figure 1a). Notably, after 1930, the errors decrease strongly for the evaluation with uncertainties added, because of a large reduction of spread in the CRUTEM5 bias ensembles (Morice et al. 2021). Before 1930, the lowest mean squared errors are observed when the data is evaluated without injected uncertainty estimates, for all scenarios. However, when uncertainties are included in the evaluation, the regression model trained with uncertainties ($\hat{\beta}^{*\,\text{GMST}}_{\text{Land:1895-06}}$) outperforms other models and the GTA estimator by approximately 15-25% before 1930 (Supplementary Figure 1c), emphasizing the importance of incorporating uncertainties in both training and evaluation phases.

The SST-based reconstruction exhibits similar trends, although the impact of different reconstruction techniques is less pronounced compared to land. Importantly, the model trained with

uncertainties consistently achieves higher performance when evaluated against data where uncertainties are considered, and it outperforms the GTA estimator as well (Supplementary Figure 1d).

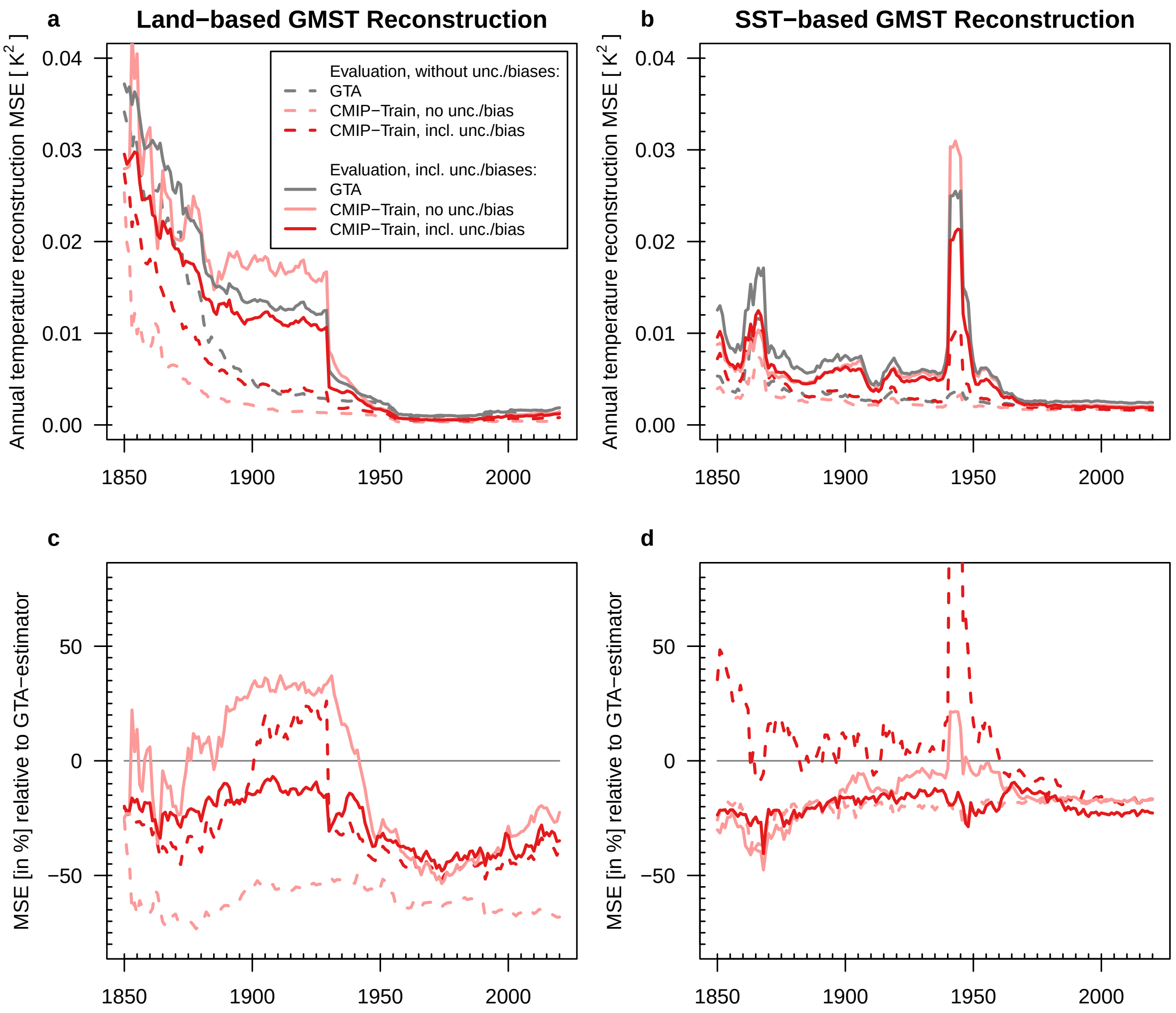

Supplementary Figure 1: **Evaluation of annual GMST reconstruction as a function of time and training setup** a. Reconstruction MSE for land-based GMST reconstruction, and c. relative to the 'Global Temperature Average' (GTA) baseline estimator similar to kriging (Cowtan et al. 2018). b. SST-based reconstruction MSE, and d. relative to the GTA baseline estimator (Cowtan et al. 2018).

2 Supplementary Reconstructions

To further evaluate our findings and reconstruction method, we provide reconstructions of different target metrics and methods across various source datasets:

Supplementary Figure 2 shows a reconstruction of GMST (similar to Figure 1 in the main text), but without adding estimates of biases and uncertainties at training time. The ocean-based GMST reconstruction is about 0.20°C colder than the land-based reconstruction in the 1900-1930 period; which is a slightly smaller discrepancy than when estimates of uncertainties and biases are added during training time (Fig. 1 in the Main Text).

Supplementary Figure 3 illustrates a reconstruction of global mean sea surface temperature (GMSST) from land and ocean, respectively.

Supplementary Figure 4 illustrates a reconstruction of global mean land surface air temperature (GMLSAT).

Supplementary Figure 5 compares our GMST reconstruction estimates from land and ocean to an additional SST-based reconstruction where the global mean SST has been removed from each grid cell, similar to (Sippel et al. 2020). While the mean-removed reconstruction reduces the overall trend as expected, it is nonetheless instructive to see that the mean-removed SST reconstruction shows higher correlation with the land-based reconstruction in the early twentieth century (Supplementary Figure 5b).

Overall, the additional reconstructions shown in Supplementary Figures 2-5 all indicate a pronounced ocean cold anomaly irrespective of the target metric or the reconstruction method. Thus, all these additional reconstructions yield very similar conclusions regarding the early twentieth century to those derived in the main text.

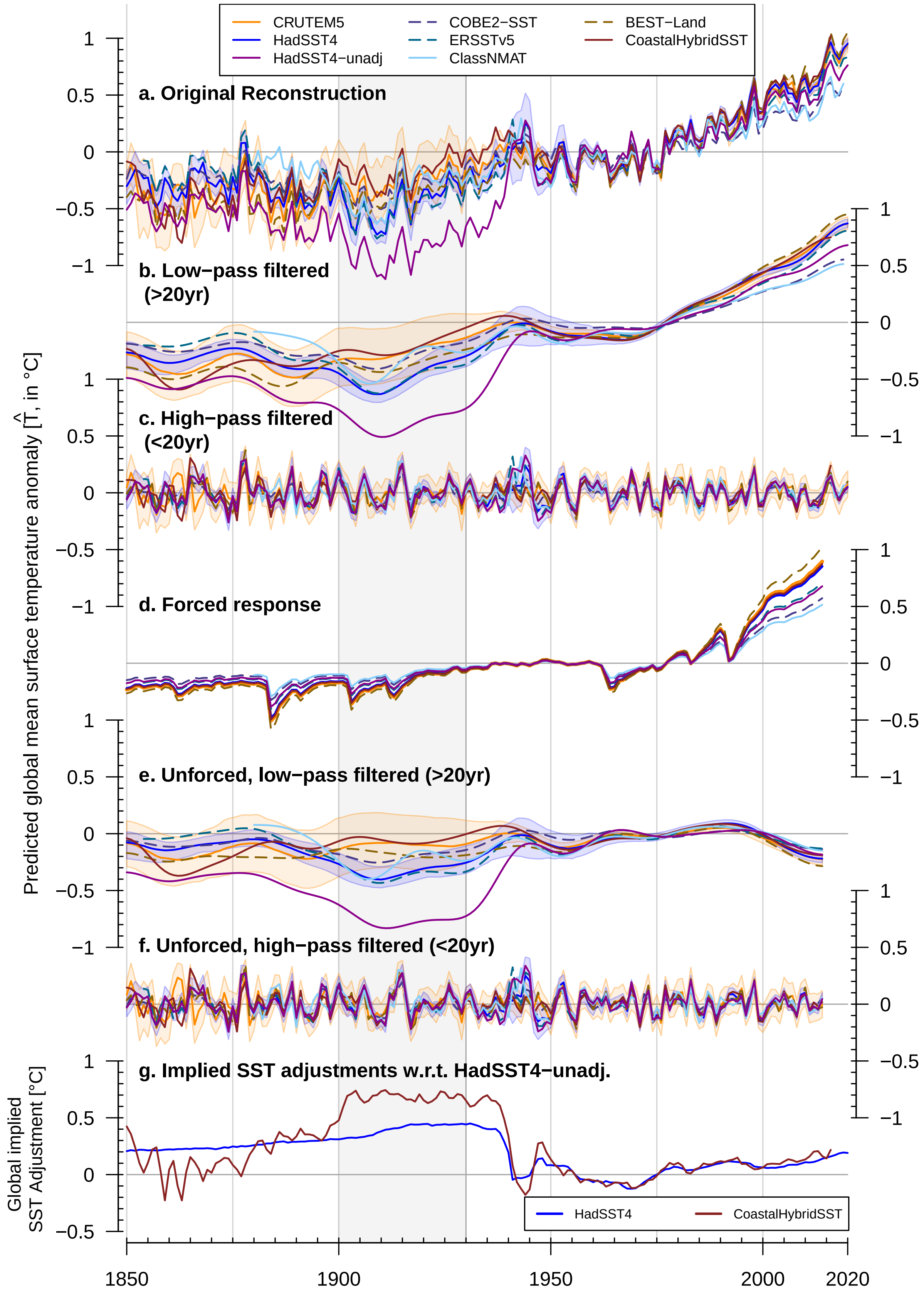

CRUTEM5
HadSST4
HadSST4−unadj
COBE2−SST
ERSSTv5
ClassNMAT
BEST−Land
CoastalHybridSST
a. Original Reconstruction
b. Low−pass filtered (>20yr)
c. High−pass filtered (<20yr)
d. Forced response
e. Unforced, low−pass filtered (>20yr)
f. Unforced, high−pass filtered (<20yr)
g. Implied SST adjustments w.r.t. HadSST4−unadj.
Predicted global mean surface temperature anomaly [T̂, in °C]
Global implied SST Adjustment [°C]
HadSST4
CoastalHybridSST
1850
1900
1950
2000
2020

Supplementary Figure 2: **Global mean surface temperature (GMST) reconstruction from the land and ocean record, but without including biases and uncertainties in the training setup.** GMST reconstructions from the SST record (HadSST4) and the land air temperature record (CRUTEM5). GMST reconstructions from HadSST4-unadj, ClassNMAT, CoastalHybridSST, BEST-Land, COBE-SST2 and ERSSTv5 are similarly derived and shown for comparison. **a.** Original GMST reconstructions, **b.** low-pass filtered reconstructions ($>$ 20-year time scale), **c.** high-pass filtered reconstructions ($<$ 20-year time scale), **d.** forced GMST response for each reconstruction, **e.** unforced, low-pass filtered reconstruction, **f.** unforced, high-pass filtered reconstruction. **g.** Implied global mean adjustments relative to unadjusted HadSST4 data, shown as the difference between the global reconstructions ($\hat{T}^{GMST}_{HadSST4} - \hat{T}^{GMST}_{HadSST4-unadj}$, and $\hat{T}^{GMST}_{CoastalHybridSST} - \hat{T}^{GMST}_{HadSST4-unadj}$). Shading represents the 95th percentile uncertainty ranges of the $\hat{T}^{GMST}_{HadSST4}$ and $\hat{T}^{GMST}_{CRUTEM5}$ reconstructions, obtained by propagating the HadSST4 and CRUTEM5 ensemble of uncertainty realizations; bold lines show the median across the ensemble.

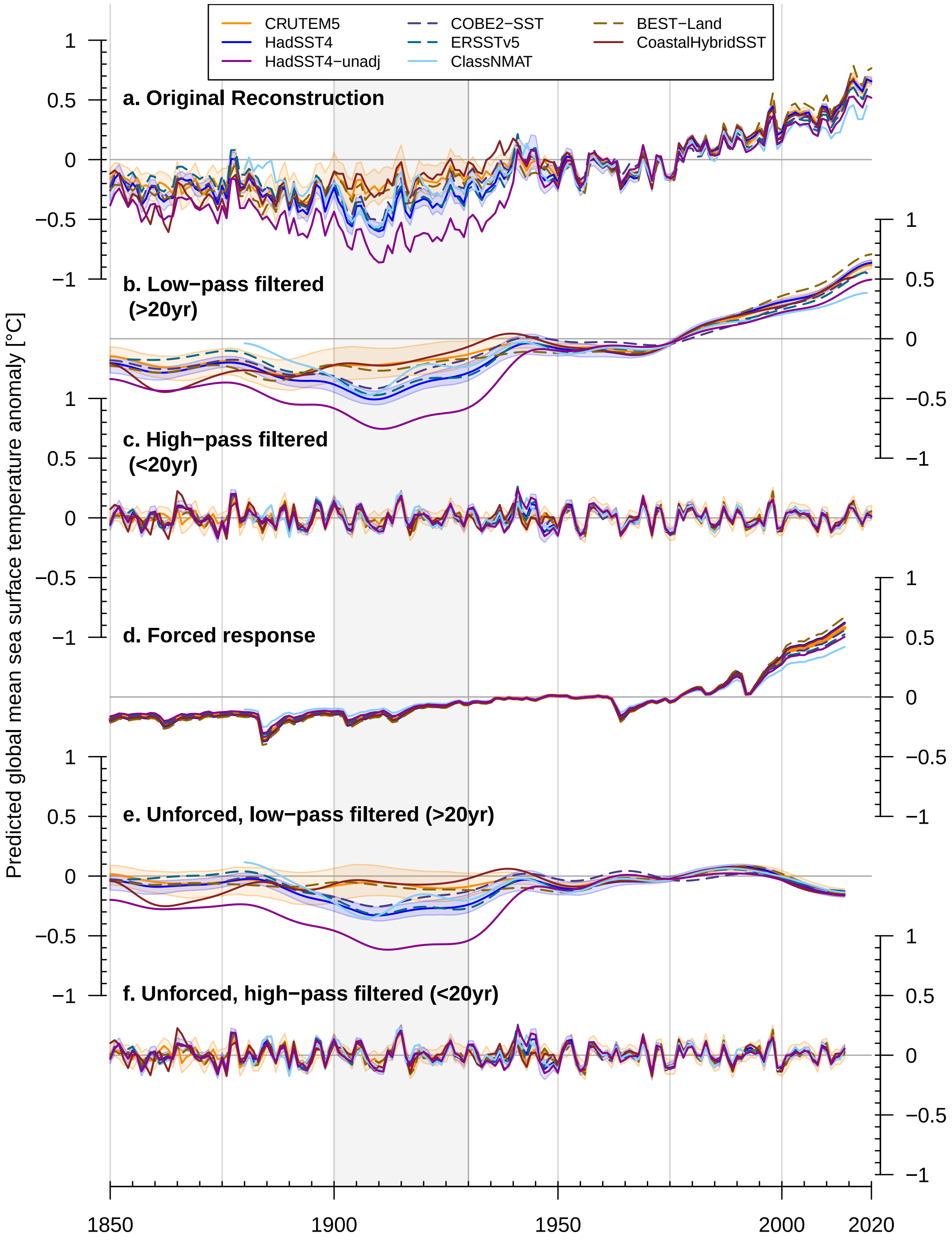

CRUTEM5
HadSST4
HadSST4−unadj
COBE2−SST
ERSSTv5
ClassNMAT
BEST−Land
CoastalHybridSST
a. Original Reconstruction
b. Low−pass filtered (>20yr)
c. High−pass filtered (<20yr)
d. Forced response
e. Unforced, low−pass filtered (>20yr)
f. Unforced, high−pass filtered (<20yr)
Predicted global mean sea surface temperature anomaly [°C]
1850
1900
1950
2000
2020

Supplementary Figure 3: **Global mean sea surface temperature (GMSST) reconstruction from the land and ocean record.** GMSST reconstructions from the SST record (HadSST4) and the land air temperature record (CRUTEM5). GMSST reconstructions from HadSST4-unadj, ClassNMAT, CoastalHybridSST, BEST-Land, COBE-SST2 and ERSSTv5 are similarly derived and shown for comparison. **a.** Original GMSST reconstructions, **b.** low-pass filtered reconstructions ($>$ 20-year time scale), **c.** high-pass filtered reconstructions ($<$ 20-year time scale), **d.** forced GMSST response for each reconstruction, **e.** unforced, low-pass filtered reconstruction, **f.** unforced, high-pass filtered reconstruction. Shading represents the 95th percentile uncertainty ranges of the $\hat{T}^{GMSST}_{HadSST4}$ and $\hat{T}^{GMSST}_{CRUTEM5}$ reconstructions, obtained by propagating the HadSST4 and CRUTEM5 ensemble of uncertainty realizations; bold lines show the median across the ensemble.

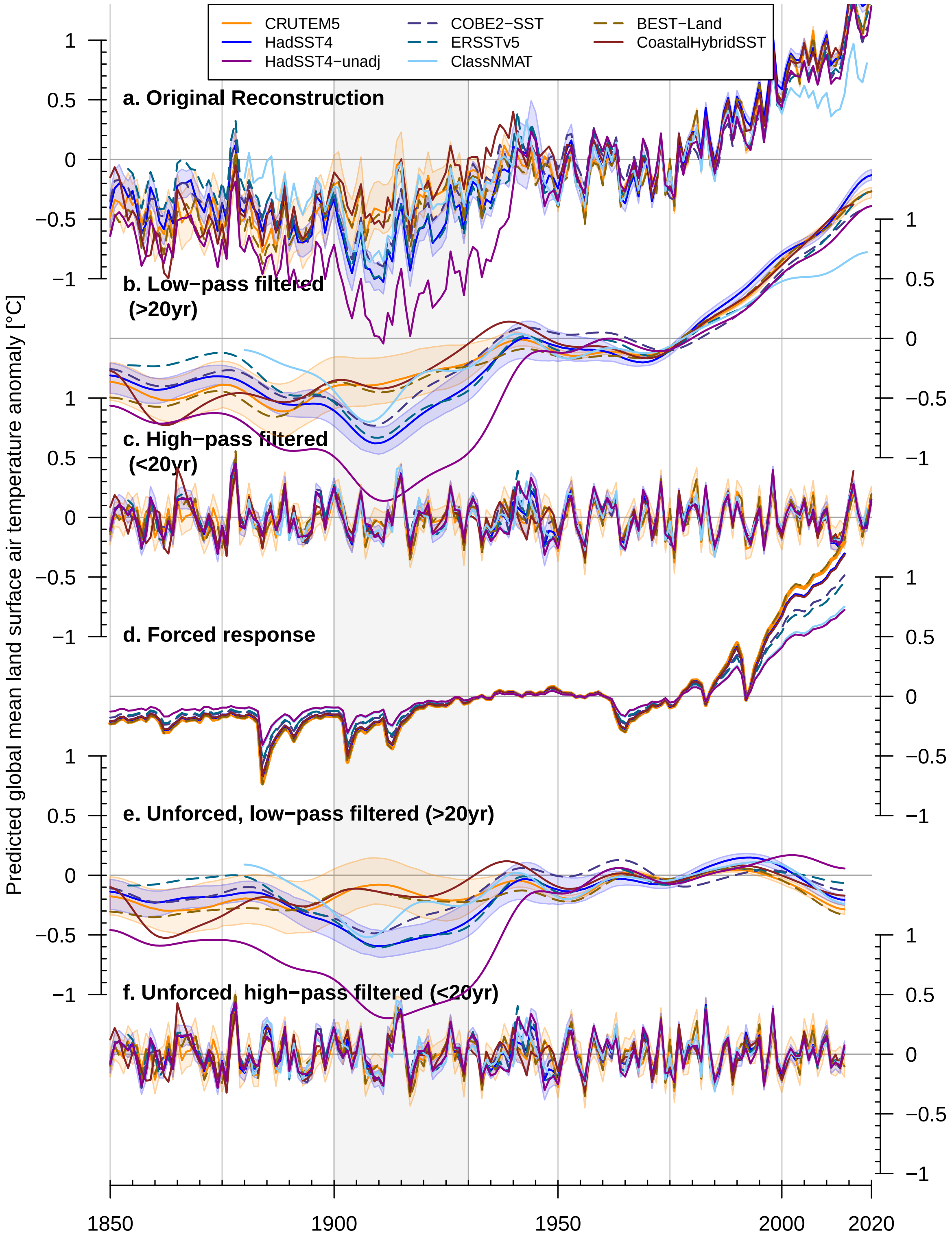

CRUTEM5
HadSST4
HadSST4−unadj
COBE2−SST
ERSSTv5
ClassNMAT
BEST−Land
CoastalHybridSST
a. Original Reconstruction
b. Low−pass filtered (>20yr)
c. High−pass filtered (<20yr)
d. Forced response
e. Unforced, low−pass filtered (>20yr)
f. Unforced, high−pass filtered (<20yr)
Predicted global mean land surface air temperature anomaly [°C]
1850
1900
1950
2000
2020

Supplementary Figure 4: **Global mean land surface air temperature (GMLSAT) reconstruction from the land and ocean record.** GMLSAT reconstructions from the SST record (HadSST4) and the land air temperature record (CRUTEM5). GMLSAT reconstructions from HadSST4-unadj, ClassNMAT, CoastalHybridSST, BEST-Land, COBE-SST2 and ERSSTv5 are similarly derived and shown for comparison. **a.** Original GMLSAT reconstructions, **b.** low-pass filtered reconstructions ($>$ 20-year time scale), **c.** high-pass filtered reconstructions ($<$ 20-year time scale), **d.** forced GMLSAT response for each reconstruction, **e.** unforced, low-pass filtered reconstruction, **f.** unforced, high-pass filtered reconstruction. Shading represents the 95th percentile uncertainty ranges of the $\hat{T}^{GMLSAT}_{HadSST4}$ and $\hat{T}^{GMLSAT}_{CRUTEM5}$ reconstructions, obtained by propagating the HadSST4 and CRUTEM5 ensemble of uncertainty realizations; bold lines show the median across the ensemble.

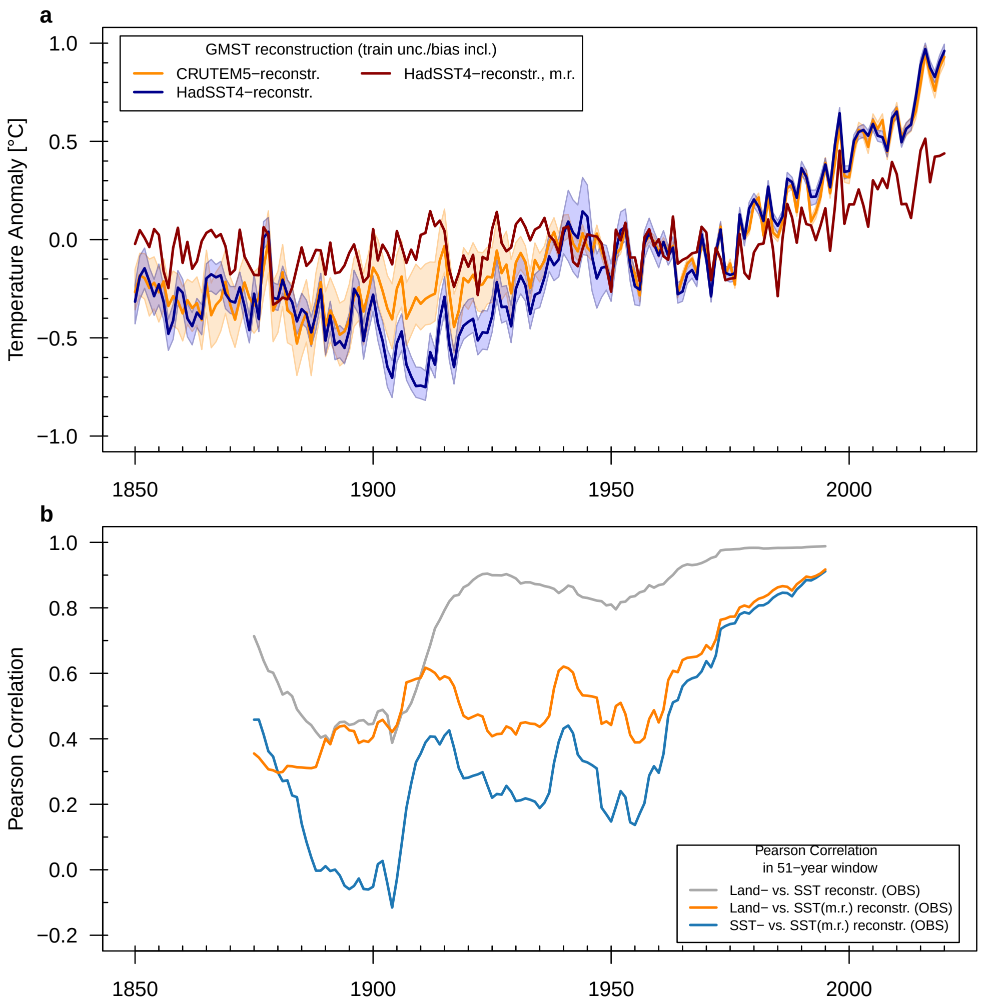

Supplementary Figure 5: **GMST reconstruction from SSTs with global mean removed. a.** this paper's land- and ocean-based GMST reconstructions along with an experimental reconstruction where the global mean is removed from the SST pattern at each time step. This way, only relative anomalies in the pattern but not the global mean is retained in the analysis. **b** shows the Pearson correlation in 51-year windows between the land- and ocean-based reconstructions, and each reconstruction with the mean-removed SST reconstruction. The mean-removed SST reconstruction shows a higher agreement with the land reconstruction (orange line) than with the original SST reconstruction (blue line).

3 Analysis of individual paleoclimate proxies from terrestrial and marine sources

In addition to the analysis of the PAGES2k and Ocean2k paleoclimate reconstructions in the main text (Tierney et al. 2015; Neukom et al. 2019), we analyse individual uncalibrated paleoclimate proxy records for their multidecadal changes between 1901-20 vs. 1871-90 (similar to the main text).

We analyzed which proxies show a positive or negative anomaly from 1901-20 compared to 1871-90. All proxy records are uncalibrated, and are shown as standardized z-scores relative to the 1871-90 reference period. The analysis reveals a mixed pattern of cooling and warming between the two periods for both land and ocean proxies (Extended Data Fig. 6; the darker the color, the greater the cooling/warming; left panels show terrestrial proxies, right panels show marine proxies). A globally coherent cooling signal, as indicated by SST datasets does not emerge in marine or terrestrial proxies. The Western Atlantic appears to be the only region where marine proxy records show predominant cooling, which is consistent with our land-based reconstruction (see Main Text). Overall, the additional analysis of individual paleoclimate proxy records supports the hypothesis that the multi-decadal discrepancy between land and ocean temperature observations in the early twentieth century may be an artefact rather than a true climatic phenomenon.

4 Supplementary analysis of ICOADS contributing sources

Relative offsets between data sources in ICOADS in the period 1870-1935 The International Comprehensive Ocean-Atmosphere Data Set Release 3 (ICOADS) (Freeman et al. 2017) is the largest archive of surface marine data spanning more than two centuries. It is the main marine data source used to generate many of the data products used in climate science, including global surface temperature (Gulev et al. 2021). To enable a comparison of different data sources, anomalies have been calculated from the SST reports in ICOADS relative to a climatology for the period 1991-2020 averaged to a 1° latitude/longitude grid (Embury et al. 2024).

Extended Data Fig. 8 shows boxplots of global annual mean anomalies for five ICOADS subsets, for the HSSTD subset and for reports originating from Germany, the Netherlands, the UK and Japan. The HSSTD subset is substantially colder than any other data source from the 1880s through to the 1910s and is the largest data source in the 1900s. The HSSTD subset is from the World Meteorological Organisation (WMO) Historical Sea Surface Temperature Data project (HSSTD) (WMO 1985). The contribution of each data source varies substantially over time (Extended Data Fig. 7) and regionally (not shown).

> ”For more than one hundred years ships of the voluntary observing fleets and more recently ocean weather ships have observed and recorded meteorological data from the oceans of the world. The Historical Sea Surface Temperature Data (HSSTD) Project was setup originally to collect all available sea surface temperature records held by the

major maritime nations for the period 1860-1960. These data were to be published in summary form for selected representative areas complemented by summary data for air temperature, surface wind speed and direction [...]

Sea surface temperature measurements by bucket only were selected for the summaries [...] Although considerable effort was made to exclude non-bucket-sea surface temperature measurements from the basic data it cannot be assumed that all unwanted measurements have been eliminated." (WMO 1985)

HSSTD reports should therefore be predominantly from buckets, as should most of the reports in this period (Kennedy et al. 2019). That the HSSTD anomalies are noticeably colder than the rest of ICOADS suggests either that there are enough unidentified reports from engine intakes in the other ICOADS data sources during this period to partly offset the expected cold bias from the bucket-derived reports (Kent and Kennedy 2021) or these data are from buckets that show a larger cold bias than other sources. After World War I the anomalies become progressively less cold.

In contrast unadjusted anomalies from German and UK data sources are warmer in each decade during the period 1800-1939. The anomalies from the different country subsets are most similar in the decade with the coldest observations, the 1910s. German, Netherlands and UK anomalies all decrease over the decades from the 1880s to the 1910s, suggesting that there are changes in bucket types or measurement protocols for each of these countries over this period

(Kent et al. 2017) with observation practices for each subset becoming more similar to those from HSSTD over this period, albeit at different rates. From the 1910s to the 1930s each subset becomes warmer with each sucessive decade, apart from the Japanese subset which is affected by data truncation during transfer to punch cards in the period 1930 to 1953 (Chan et al. 2019). This is consistent with either improved protocols for bucket measurements leading to smaller cold biases or to an increasing contribution of measurements from engine intakes, or most likely both.

Investigation into the source of the HSSTD records in ICOADS ICOADS has been constructed from a variety of different data sources using a procedure called "dupelim" (`https://icoads.noaa.gov/Release_1/suppK.html`) to identify duplicates (different versions of the same original observation) among those data sources. In earlier releases of ICOADS (prior to Release 2.5) the discarded duplicates were excluded from the archive which means it is not possible to compare different versions of the same record that have arrived in ICOADS through different routes. The ICOADS DCK indicator gives information on the source of reports. The term DCK originates from the decks of punch cards that were the sources of data used prior to when ICOADS (then COADS) was assembled. Differences can arise between records from different sources for a variety of reasons: conversions from local standard time to UTC may have been performed differently; elements may be dropped, rounded or truncated due to restrictions in the formats used; codes and derived variables may be subject to different conversions; there may be systematic errors in coding or translations; or transcription problems affecting individual reports. This means that the comparisons between sources to identify possible duplicates is not an exact process and needs to employ tolerances. Whilst we have basic information on

the matches that were made and the characteristics of the data (for the 1800s here: `https://icoads.noaa.gov/e-doc/other/dupelim_sum_1800s` and for 1900 to 1945 here: `https://icoads.noaa.gov/e-doc/other/dupelim_sum_1900s`) it is not possible to make more detailed comparisons using this information.

Supplementary Figure 6 shows the output of the dupelim summary for the period 1800 to 1899 visualised as a chord diagram for data sources identified by their ”DCK” indicator (Smith et al. 2022) or for the HSSTD grouping. Each chord is coloured according to the source selected for each pair, the colours around the edge represent the different sources. In the 1800s most of the data from HSST (red sector) get replaced with data from ICOADS DCKs 192, 193, 194 and 201 (data from the German, Netherlands and UK archives (see key and (Smith et al. 2022)). The right hand panel shows the same information but with the log of the contributions to emphasise the smaller contributions. Figure 7 presents the same information for the period 1900 to 1945, with DCK contributing to fewer than 200k pairs combined as ”other”. For this period more of the HSST data is retained. As this information on DCK matches is extracted from summaries provided by ICOADS it is not possible to examine breakdowns of this information for different periods or regions.

The characteristics of HSSTD are different from the other main sources in ICOADS. Because HSSTD were selected to be from buckets only, observations from ICOADS that were paired with HSSTD are assigned the measurement method ”implied bucket”. Stratifying the data for Germany, the Netherlands and the UK according to whether a match with HSSTD had been identified showed

little difference between the reports for each country depending on this assignment. This suggests that the reason for the relatively cold anomalies in HSSTD is not due to unidentified engine intake observations, and that the remaining HSSTD reports in ICOADS are a distinct data source using bucket types and measurement protocols that gave a larger than typical heat loss. US observation sources are largely absent in this period, so it can be hypothesized that the HSSTD are from the missing US national archive. The observing instructions for the US published in 1906 (and also in 1910), describes a sampling protocol likely to lead to relative large cold biases in the observations:

> ”The water whose temperature is taken should be drawn from a depth of 3 feet below the surface, the bucket in which it is drawn being weighted in order to sink it. The bulb of the thermometer should remain immersed in the water at least three minutes before reading, and the reading should be made with the bulb immersed.” (Page 1906)

In 1925 the US instructions (*Instructions to Marine Meteorological Observers* 1925) remain the same, but also notes the desirability for a shorter gap of about 1 minute between sampling and measurement and recommended active stirring. A special brass sampling container is pictured but also suggests vertical stiffening for the ordinary canvas bucket. By 1929 (*Instructions to Marine Meteorological Observers* 1929) the instructions are similar but with further encouragement toward shorter measurement periods especially when the wet bulb depression is large and that measurement should be made out of both the wind and the sun.

By 1938 the US instructions provide seven steps for making accurate SST measurements using buckets, emphasizing the need to make the measurement as quickly as it is safe to do (*Instructions to Marine Meteorological Observers* 1938). The benefits of stiffening of canvas buckets is mentioned and the picture of the brass sampler is not included. The instructions describe the engine intake method as simpler, and stresses that the method used should be chosen to be that expected to be more accurate and that the method used should be recorded. This is again consistent with a reducing cold anomaly for the HSSTD over the period 1920 onwards (Extended Data Fig. 8).

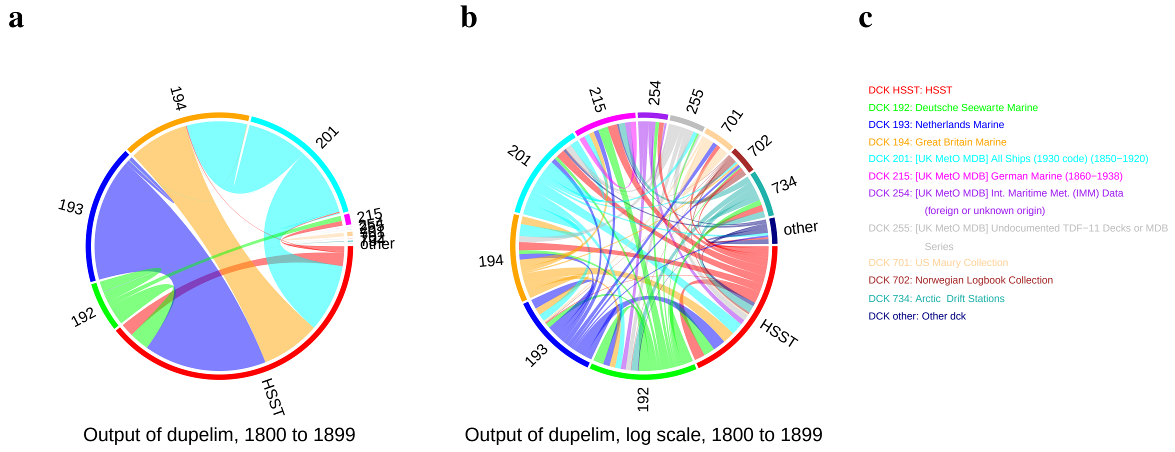

Supplementary Figure 6: **Dupelim output information from ICOADS Release 1, 1800-1899**. Segments of the circle are coloured by data source (ICOADS DCK or HSST) and the connecting chord is colored by the retained source. Data sources contributing to fewer than 1000 matches have been combined into a single category (other). **a** Visualisation of number of pairs; **b** log of number of pairs; **c** key to data sources.

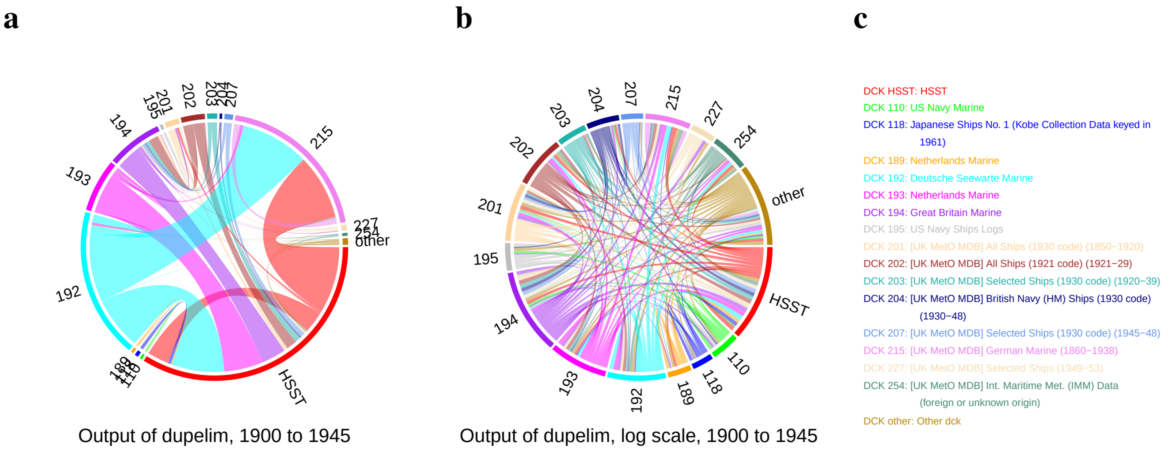

Supplementary Figure 7: **Dupelim output information from ICOADS Release 1, for 1900-1945**. Data sources contributing to fewer than 200k matches have been combined into a single category (other). **a** Visualisation of number of pairs; **b** log of number of pairs; **c** key to data sources.

5 Ocean warming constrained by land warming and paleoclimate reconstructions using 50-year trends

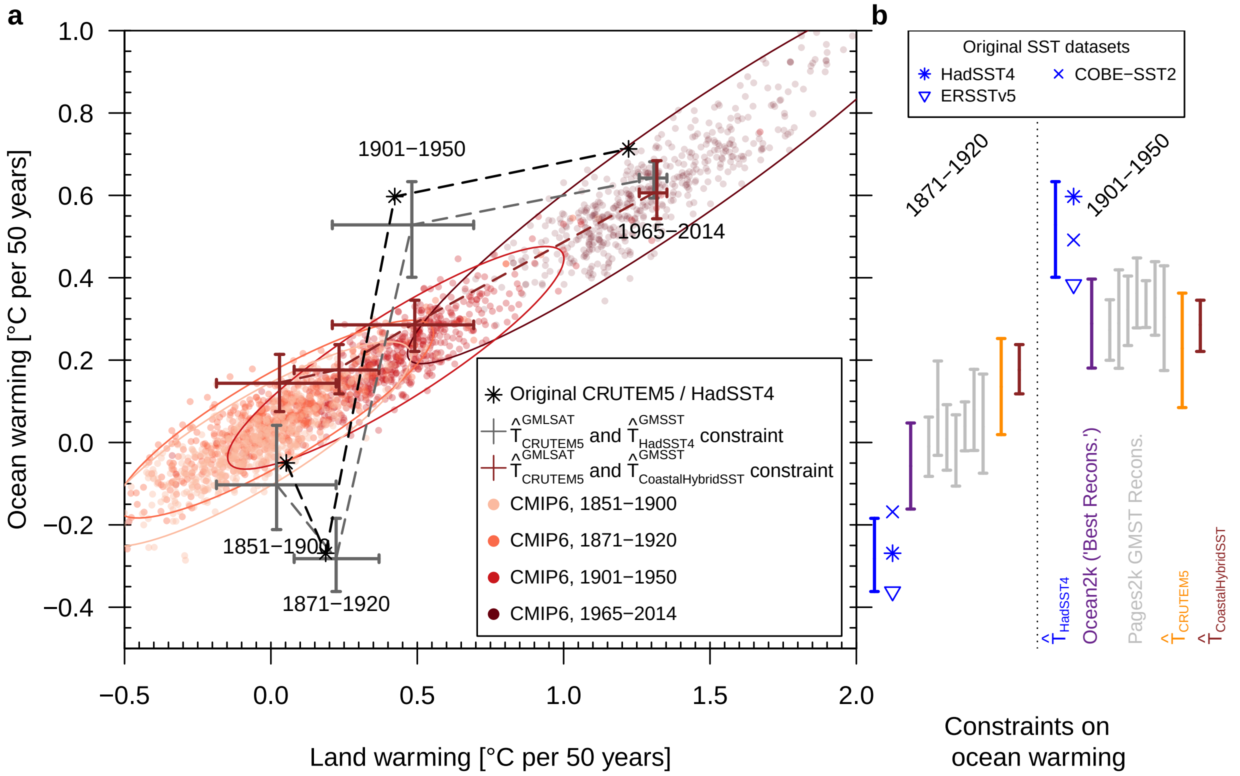

Supplementary Figure 8: **Ocean warming constrained by land warming and paleoclimate reconstructions, shown for 50-year trends.** **a**. Land and ocean warming on multi-decadal time scales is closely linked across CMIP6 models (trends over 50 years for different historical periods). **b**. Constraints from land air temperature (CRUTEM5 and Coastal Hybrid SST) and paleoclimate reconstructions (PAGES 2k and Ocean 2k) show reduced ocean cooling in the 1871-1920 period due to a less pronounced early twentieth century cold anomaly. This period is followed by more moderate 1901-1950 warming compared to HadSST4 data.

6 Supplementary Tables

Supplementary Table 1: Overview of global mean temperature reconstructions by source and training dataset.

Target metrics	Source dataset	Training dataset + mask	Training uncertainties	Further notes
GMST, GMSST, GMLSAT, IO, WP, WA	CRUTEM5 (LSAT)	CMIP6 LSAT & CRUTEM5 mask	CRUTEM5	Standard land-based reconstruction
GMST, GMSST, GMLSAT	Berkeley Earth Land (LSAT)	CMIP6 LSAT & CRUTEM5 mask	CRUTEM5	-
GMST, GMSST, GMLSAT, TMSST	HadSST4 (SST)	CMIP6 SST & HadSST4 mask	HadSST4	Standard ocean-based reconstruction
GMST, GMSST, GMLSAT	COBE-SST2 (SST)	CMIP6 SST & HadSST4 mask	HadSST4	-
GMST, GMSST, GMLSAT	ERSST5 (SST)	CMIP6 SST & HadSST4 mask	HadSST4	-
GMST, GMSST, GMLSAT	HadSST4-unadj	CMIP6 SST & HadSST4 mask	HadSST4	Unadjusted HadSST4 dataset
GMST, GMSST, GMLSAT	HadSST4-MR (SST)	CMIP6-MR SST & HadSST4 mask	HadSST4	global mean removed from each grid cell for each time step
GMST, GMSST, GMLSAT	Cowtan-Hybrid36 (SST)	CMIP6 SST & HadSST3 mask	HadSST3	SST dataset with coastal corrections to match LSATs
GMST, GMSST, GMLSAT	ClassNMAT (NMAT)	CMIP6 MAT & ClassNMAT mask	ClassNMAT	Reconstruction based on night-time marine air temperatures

Supplementary Table 2: Overview of gridded observational datasets used for reconstructions

Dataset short name	Long name	Variable	Reference	URL	Notes
CRUTEM 5.0.1.0	Climatic Research Unit temperature version 5	LSAT	Osborn et al. (2021)	`https://www.metoffice.gov.uk/hadobs/crutem5/data/CRUTEM.5.0.1.0/download.html`	incl. error estimates (CRUTEM5) and bias ensemble extracted from HadCRUT5
HadSST 4.0.1.0	Met Office Hadley Centre SST data set version 4	SST	Kennedy et al. (2019)	`https://www.metoffice.gov.uk/hadobs/hadsst4/data/download.html`	incl. error estimates and bias ensemble from HadSST4
ClassNMAT 1.0.0.0	Climate Linked Atlantic Sector Science (CLASS) night-time marine air temperature	NMAT	Cornes et al. (2020)	`https://catalogue.ceda.ac.uk/uuid/5bbf48b128bd488dbb10a56111feb36a`	incl. ClassNMAT error estimates
BEST-Land	Berkeley Earth Land	LSAT	Rohde and Hausfather (2020)	`https://berkeleyearth.org/data/`	-
ERSSTv5	Extended Reconstructed Sea Surface Temperature, Version 5	SST	Huang et al. (2017)	`https://psl.noaa.gov/data/gridded/data.noaa.ersst.v5.html`	-
COBE-SST2	Centennial In Situ Observation Based Estimates of the Variability of SST and Marine Meteorological Variables (COBE)	SST	Hirahara, Ishii, and Fukuda (2014)	`https://psl.noaa.gov/data/gridded/data.cobe2.html`	-
Coastal-HybridSST	Coastal SST corrected dataset	SST	Cowtan, Rohde, and Hausfather (2018)	`https://www-users.york.ac.uk/~kdc3/papers/evaluating2017/`	-

Supplementary Table 3: Overview of GMST datasets and paleoclimate reconstructions

Dataset short name	Long name	Variable	Reference	URL	Notes
HadCRUT5	Hadley Centre/Climatic Research Unit global surface temperature dataset	GMST	Morice et al. (2021)	https://www.metoffice.gov.uk/hadobs/crutem5/data/CRUTEM.5.0.1.0/download.html	-
BEST	Berkeley Earth Global Monthly Land + Ocean	GMST	Rohde and Hausfather (2020)	https://berkeleyearth.org/data/	-
CW2014	Cowtan and Way kriging-interpolated HadCRUT4	GMST	Cowtan and Way (2014)	https://www-users.york.ac.uk/~kdc3/papers/coverage2013/	-
CW2014-COBE2	Cowtan and Way kriging-interpolated COBE2	GMST	Cowtan and Way (2014)	https://www-users.york.ac.uk/~kdc3/papers/coverage2013/	-
JMA-GMST	Japanese Meteorological Agency Global Average Surface Temperature Anomalies	GMST	Hirahara, Ishii, and Fukuda (2014)	https://ds.data.jma.go.jp/tcc/tcc/products/gwp/temp/ann_wld.html	-
NOAA GlobTemp 5.1	NOAA Merged Land Ocean Global Surface Temperature Analysis	GMST	Vose et al. (2021)	https://www.ncei.noaa.gov/products/land-based-station/noaa-global-temp	-
NASA-GISTEMP	GISS Surface Temperature Analysis 4	GMST	Lenssen et al. (2019)	https://data.giss.nasa.gov/gistemp/	-
Pages 2k GMST	PAGES 2k multi-proxy GMST reconstructions	GMST (from proxies)	Emile-Geay et al. (2017) and Neukom et al. (2019)	https://www.ncei.noaa.gov/access/paleo-search/study/26872	-
Ocean 2k	Ocean 2k Tropical sea surface temperature reconstructions	GMST	Tierney et al. (2015)	https://www.ncei.noaa.gov/access/paleo-search/study/17955	-

Supplementary Table 4: Overview of CMIP6 models used in the analysis.

Model name	Model abbrev.[a]	# Ens. Members in Training	# Ens. Members in Analysis
ACCESS-CM2	ACC	3	5
ACCESS-ESM1-5	ACC	3	40
AWI-CM-1-1-MR	AWI	0	5
AWI-ESM-1-1-LR	AWI	0	1
BCC-CSM2-MR	BCC	0	3
BCC-ESM1	BCC	3	3
CAMS-CSM1-0	CAM	3	3
CESM2-FV2	CES	2	3
CESM2-WACCM-FV2	CES	2	3
CESM2-WACCM	CES	2	3
CESM2	CES	2	11
CIESM	CIE	0	3
CMCC-CM2-HR4	CMC	0	1
CMCC-CM2-SR5	CMC	3	11
CMCC-ESM2	CMC	0	1
CNRM-CM6-1-HR	CNR	0	1
CNRM-CM6-1	CNR	3	30
CNRM-ESM2-1	CNR	3	11
CanESM5-CanOE	Can	3	3
CanESM5	Can	3	65
E3SM-1-0	E3S	3	4
E3SM-1-1-ECA	E3S	0	1
E3SM-1-1	E3S	0	1
EC-Earth3-AerChem	EC-	0	1
EC-Earth3-Veg-LR	EC-	2	3
EC-Earth3-Veg	EC-	2	8
EC-Earth3	EC-	2	23
FGOALS-f3-L	FGO	3	3
FGOALS-g3	FGO	3	6
FIO-ESM-2-0	FIO	0	3
GFDL-ESM4	GFD	3	3
GISS-E2-1-G-CC	GIS	0	1
GISS-E2-1-G	GIS	3	40
GISS-E2-1-H	GIS	3	25
GISS-E2-2-G	GIS	0	11
GISS-E2-2-H	GIS	0	5
HadGEM3-GC31-LL	Had	2	5
HadGEM3-GC31-MM	Had	2	4
INM-CM4-8	INM	0	1
INM-CM5-0	INM	3	10
IPSL-CM6A-LR-INCA	IPS	0	1
IPSL-CM6A-LR	IPS	3	33
KACE-1-0-G	KAC	0	3
MIROC-ES2H	MIR	2	3
MIROC-ES2L	MIR	2	31
MIROC6	MIR	2	50
MPI-ESM-1-2-HAM	MPI	2	3
MPI-ESM1-2-HR	MPI	2	10
MPI-ESM1-2-LR	MPI	2	31
MRI-ESM2-0	MRI	3	12
NESM3	NES	0	5
NorCPM1	Nor	2	30
NorESM2-LM	Nor	2	3
NorESM2-MM	Nor	2	3
SAM0-UNICON	SAM	0	1
TaiESM1	Tai	0	1
UKESM1-0-LL	UKE	3	17
UKESM1-1-LL	UKE	0	1
Total		93	602

[a] Model abbreviation indicates the models that stem from the same model variant and are used in the training step.

References

Chan, D. et al. (2019). “Correcting datasets leads to more homogeneous early-twentieth-century sea surface warming”. In: *Nature* 571.7765, pp. 393–397.

Cornes, R. C. et al. (2020). “CLASSnmat: A global night marine air temperature data set, 1880–2019”. In: *Geoscience Data Journal* 7.2, pp. 170–184.

Cowtan, K., R. Rohde, and Z. Hausfather (2018). “Evaluating biases in sea surface temperature records using coastal weather stations”. In: *Quarterly Journal of the Royal Meteorological Society* 144.712, pp. 670–681.

Cowtan, K. and R. G. Way (2014). “Coverage bias in the HadCRUT4 temperature series and its impact on recent temperature trends”. In: *Quarterly Journal of the Royal Meteorological Society* 140.683, pp. 1935–1944.

Cowtan, K. et al. (2018). “Statistical analysis of coverage error in simple global temperature estimators”. In: *Dynamics and Statistics of the Climate System* 3.1, dzy003.

Embury, O. et al. (2024). “Satellite-based time-series of sea-surface temperature since 1980 for climate applications”. In: *Scientific Data* 11.1.

Emile-Geay, J. et al. (2017). “A global multiproxy database for temperature reconstructions of the Common Era”. In: *Scientific Data* 4.1, p. 170088.

Freeman, E. et al. (2017). “ICOADS Release 3.0: a major update to the historical marine climate record”. In: *International Journal of Climatology* 37.5, pp. 2211–2232.

Gulev, S. K. et al. (2021). *Changing state of the climate system. In Climate Change 2021: The Physical Science Basis. Contribution of Working Group I to the Sixth Assessment Report of the Intergovernmental Panel on Climate Change*. IPCC Sixth Assessment Report.

Hirahara, S., M. Ishii, and Y. Fukuda (2014). “Centennial-Scale Sea Surface Temperature Analysis and Its Uncertainty”. In: *Journal of Climate* 27.1, pp. 57–75.

Huang, B. et al. (2017). “Extended Reconstructed Sea Surface Temperature, Version 5 (ERSSTv5): Upgrades, Validations, and Intercomparisons”. In: *Journal of Climate* 30.20, pp. 8179–8205.

Instructions to Marine Meteorological Observers (1925). Tech. rep. Circular M, Fourth Edition. Washington, US: US Weather Bureau.

Instructions to Marine Meteorological Observers (1929). Tech. rep. Circular M, Fifth Edition. Washington, US: US Weather Bureau.

Instructions to Marine Meteorological Observers (1938). Tech. rep. Circular M, Sixth Edition. Washington, US: US Weather Bureau.

Kennedy, J. J. et al. (2019). “An Ensemble Data Set of Sea Surface Temperature Change From 1850: The Met Office Hadley Centre HadSST.4.0.0.0 Data Set”. In: *Journal of Geophysical Research: Atmospheres* 124.14, pp. 7719–7763.

Kent, E. C. and J. J. Kennedy (2021). “Historical Estimates of Surface Marine Temperatures”. In: *Annual Review of Marine Science* 13.1, pp. 283–311.

Kent, E. C. et al. (2017). “A Call for New Approaches to Quantifying Biases in Observations of Sea Surface Temperature”. In: *Bulletin of the American Meteorological Society* 98.8, pp. 1601–1616.

Lenssen, N. J. L. et al. (2019). “Improvements in the GISTEMP Uncertainty Model”. In: *Journal of Geophysical Research: Atmospheres* 124.12, pp. 6307–6326.

Morice, C. P. et al. (2021). “An Updated Assessment of Near-Surface Temperature Change From 1850: The HadCRUT5 Data Set”. In: *Journal of Geophysical Research: Atmospheres* 126.3, e2019JD032361.

Neukom, R. et al. (2019). “Consistent multidecadal variability in global temperature reconstructions and simulations over the Common Era”. In: *Nature Geoscience* 12.8, pp. 643–649.

Osborn, T. J. et al. (2021). “Land Surface Air Temperature Variations Across the Globe Updated to 2019: The CRUTEM5 Data Set”. In: *Journal of Geophysical Research: Atmospheres* 126.2, e2019JD032352.

Page, J. (1906). *Instructions to the Marine Meteorological Observers of the U. S. Weather Bureau*. Tech. rep. Washington, US: US Weather Bureau.

Rohde, R. A. and Z. Hausfather (2020). “The Berkeley Earth Land/Ocean Temperature Record”. In: *Earth System Science Data* 12.4, pp. 3469–3479.

Sippel, S. et al. (2020). “Climate change now detectable from any single day of weather at global scale”. In: *Nature Climate Change* 10.1, pp. 35–41.

Smith, S. R. et al. (2022). *The International Maritime Meteorological Archive (IMMA) Format*. Tech. rep. ICOADS.

Tierney, J. E. et al. (2015). “Tropical sea surface temperatures for the past four centuries reconstructed from coral archives”. In: *Paleoceanography* 30.3, pp. 226–252.

Vose, R. S. et al. (2021). “Implementing Full Spatial Coverage in NOAA’s Global Temperature Analysis”. In: *Geophysical Research Letters* 48.4, e2020GL090873.

WMO (1985). *Users’s Guide to the Data and Summaries of the Historical Sea Surface Temperature Data Project*. Tech. rep. WMO/TD-No. 36. Geneva, Switzerland: Secretariat of the World Meteorological Organization.
